# Variable Stromal Periductular Expression of CD34 and Smooth Muscle Actin (SMA) in Intraductal Carcinoma of the Breast

**DOI:** 10.1371/journal.pone.0057773

**Published:** 2013-03-01

**Authors:** Xavier Catteau, Philippe Simon, Michel Vanhaeverbeek, Jean-Christophe Noël

**Affiliations:** 1 Institute of Pathology and Genetics, Gosselies, Belgium; 2 Unit of Gynaecology, Erasme’s University Hospital-ULB, Brussels, Belgium; 3 Laboratory of Experimental Medicine, Centre Hospitalo-Universitaire de Charleroi, André Vésale Hospital-Université Libre de Bruxelles, Montigny-Le-Tilleul, Belgium; 4 Unit of Gynaecopathology, Pathology Department, Erasme’s University Hospital- Université Libre de Bruxelles, Brussels, Belgium; Health Canada, Canada

## Abstract

In breast carcinoma, the stromal loss of CD34 expression and acquisition of SMA myofibroblastic features may constitute a prerequisite for tumor invasiveness. However, this hypothesis remains controversial, with some authors describing the loss of CD34 fibrocytes in the absence of SMA myofibroblastic-like cells in the stroma of invasive carcinoma. Others have also described the disappearance of CD34 fibrocytes from in situ carcinoma. To clarify this issue, we compared the distribution of CD34 fibrocytes and SMA reactive myofibroblasts between stromal areas of tumor-free mammary tissue, ductal carcinoma in situ (DCIS) and invasive ductal carcinoma (IDC). In addition to 28 IDC, 300 normal duct–lobular units and 600 ducts with DCIS (158 low-grade, 266 intermediate, and 176 high-grade) were scored. The relationships between staining patterns and different histological features (grade of DCIS and presence or absence of necrosis) were compared. Loss of CD34 expression and acquisition of SMA expression were more frequent in high-grade in situ lesions than in intermediate and low-grade lesions (p<0.001). When necrosis was found in association with grade 2 or 3 DCIS, the decrease in CD34 expression was higher than in lesions without necrosis and that independently of the grade of DCIS (p<0.05). Necrosis did not appear to play a significant role in the expression of SMA (p = 0.35). In all cases, the stroma of invasive carcinomas showed a complete loss of CD34 fibrocytes. Future research on both CD34 fibrocytes and mechanisms stromal changes are essential in the future and may potentially lead to new treatment approaches.

## Introduction

Epithelial-mesenchymal interactions are critical for normal mammary gland development and for breast tumorigenesis [1]. In vivo and in vitro studies have demonstrated that the extracellular matrix (ECM) molecules and cells that compose the microenvironment modulate tissue-specificity in the normal breast, as well as the growth, survival, polarity, and invasive behavior of breast cancer cells [2], [3].

Normal mammary stroma harbor huge numbers of CD34 fibrocytes, which can first be detected during the 10^th^ gestational week. In this developmental phase they make up the majority of stromal cells [4].

In breast carcinoma, recent data suggest that CD34 fibrocytes undergo morphologic and phenotypic alterations characterized by the adoption of a plump myofibroblast-like appearance and loss of CD34 expression, accompanied by the acquisition of α-smooth muscle actin (SMA) expression [5]. The stromal loss of CD34 expression and acquisition of SMA myofibroblastic features may constitute a prerequisite for tumor invasiveness [6], [7]. However, this hypothesis remains controversial, with some authors describing the loss of CD34 fibrocytes in the absence of SMA myofibroblastic-like cells in the stroma of invasive carcinoma. Others have also described the disappearance of CD34 fibrocytes from in situ carcinoma [8]–[10]. In order to clarify this issue, we respectively analyzed the presence of both CD34 fibrocytes and SMA myofibroblasts in the peritumoral stroma of ductal carcinoma in situ (DCIS) and invasive ductal carcinoma (IDC).

## Materials and Methods

The study protocol was approved by the institutional ethics and research review boards at Erasme Hospital. People sign a written informed consent to admission to the hospital. Consent requires that physicians have the right to use the surplus biological material. The material that has not been used for diagnosis can be used for research. Consent has been established by the local ethics committee and is in accordance with Belgian and International law. We used a computer database from the Department of Pathology to identify 48 consecutive patients diagnosed between January 2010 and June 2012. All patients were female. The present retrospective study included 20 pure DCIS, 12 DCIS associated with IDC and 16 pure IDC (biopsy or surgical specimens). For each case of ductal carcinoma in situ (DCIS), all the foci were graded and analyzed separately representing a total of 600 foci of DCIS. In the majority of cases (27/32), all the foci of DCIS showed the same grade, but in 5 cases, a mixture of different grades was observed (in 3 cases a mixture of grades 2 and 3, in one case a mixture of grades 1 and 2 and in one case a mixture of grades 1 and 3).

### Immunohistochemistry

The specimens were fixed in histology-grade 4% buffered formalin. Paraffin sections were stained with hematoxylin and eosin and immunohistochemical detection was performed according to the manufacturer’s protocols (Table 1). We used a fully automated immunohistochemical system (Bond Vision Biosystems).

**Table 1:** Antibodies used in this study.

Antigen	Clone	Dilution	Antigen retrieval	Source
CD 34	QBEnd-10	1/500	H1 (30)/30	Menarini
CD 31	1A10	1/1000	H2 (30)/30	Menarini
α-SMA	αSM-1	1/100	−/30	Menarini
p 63	7JUL	1/200	H2 (20)/30	Leica Biosystems

### Semi-quantitative Assessment of Immunohistochemistry

We compared the distribution of CD34 fibrocytes and SMA reactive myofibroblasts between stromal areas located within the tumor with areas of tumor-free mammary tissue surrounding the carcinoma. Because endothelial cells expressed CD34 and myoepithelial cells expressed SMA, CD31 and p63 were also assessed to facilitate the semi-quantitative evaluation of CD34 and SMA in stromal tissue. The immunoreactivity of CD34 and SMA was assessed semi-quantitatively in the tumor-free tissue and the tumor. The percentage of stromal cells expressing each antigen was graded as “0”, “+”, “++” and “+++” when up to 5%, more than 5% and up to 25%, more than 25% and up to 50% or more than 50% of stromal cells, disclosed immunoreactivity, respectively. Percentages were assessed by two independent observers, assuming that a high-power microscopic field (objective 40×, microscopic magnification: ×400) harbored 100 stromal cells (range: 75–150). Because many of the sections contained ducts and lobules showing a range of histopathological features, the relationship between staining patterns and pathology was interpreted for each duct-lobular unit. Thus, in addition to 28 IDC, 300 normal duct-lobular units and 600 ducts with DCIS (158 low grade, 266 intermediate, and 176 high grade) were scored. When foci of DCIS were associated with invasive lesions, we considered them separately at the periphery of invasive areas and at the center of invasive areas. DCIS lesions located in the centers of invasive areas were not included in statistical tests. We also took into account the presence or absence of necrosis associated with grade 2 or 3 carcinoma in situ. The relationship between the staining patterns and different histological features (grade of DCIS and presence or absence of necrosis) was compared using a Chi-squared test, and a p-value <0.05 was considered statistically significant.

## Results

### Normal Breast

**Figure 1:**
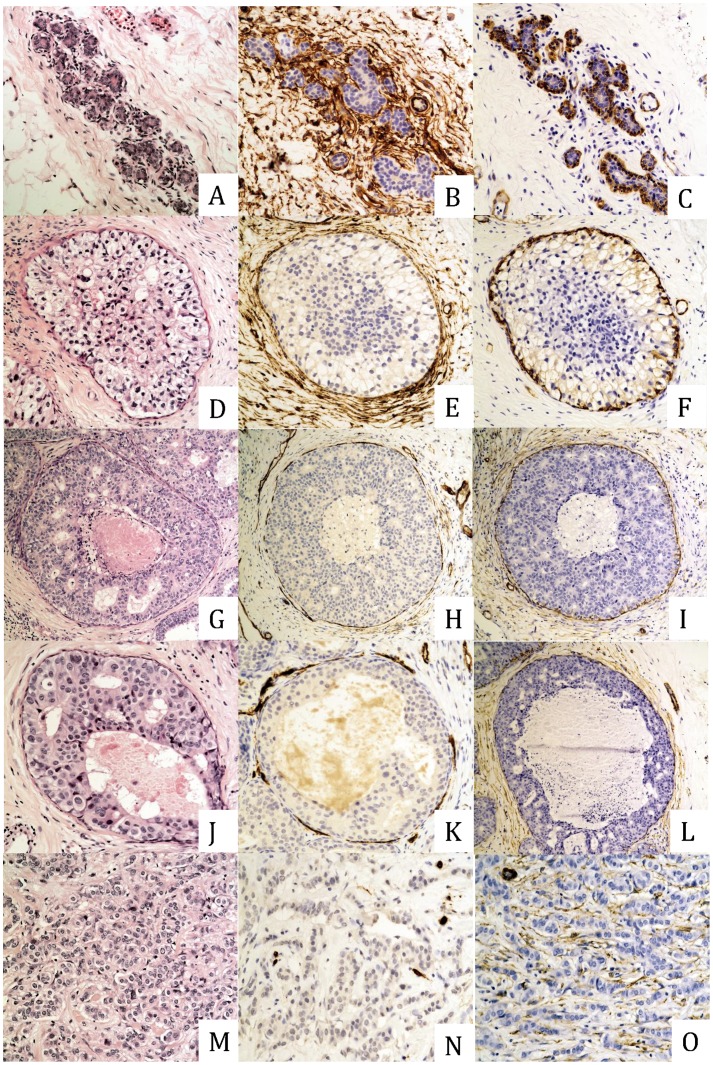
CD34 and SMA expression in normal breast tissue, ductal carcinoma in situ and invasive ductal carcinoma. A: Normal breast ducts (H&E staining). B: Diffuse CD34 expression within the periductal stroma of normal ducts. C: Absence of SMA expression within the periductal stroma of normal ducts. D: Focus of low-grade DCIS (H&E staining). E: Preserved CD34 expression around foci of low-grade DCIS. F: Absence of SMA expression within the periductal stroma of low-grade DCIS lesions. G: Focus of intermediate-grade DCIS (H&E staining). H: Decrease in CD34 expression within the periductal stroma of intermediate-grade DCIS foci. I: Emergence of SMA expression within the periductal stroma of intermediate-grade DCIS foci. J: Focus of high-grade DCIS (H&E staining). K: Strong decrease or loss of CD34 expression within the periductal stroma of high-grade DCIS foci. L: Emergence of a large contingent of SMA-positive stromal cells around high-grade DCIS foci. M: Focus of invasive ductal carcinoma (H&E staining). N: Complete loss of CD34 expression within the stroma of invasive foci. O: Strong and diffuse expression of SMA within the stroma of invasive foci. DCIS: ductal carcinoma in situ; SMA: alpha smooth muscle antigen.

In normal mammary tissue, muscular blood vessels, glandular ducts, and acinii were surrounded by a dense concentric network of CD34 fibrocytes. Slight CD34 staining was noted on small-caliber blood vessels within the stroma, and was confirmed by the expression of the vascular antigen CD31. Fibrocytes were predominantly found in the intralobular parenchyma, and the extralobular stroma harbored few CD34 fibrocytes, which mainly surrounded thick-walled arteries. These cells revealed slender elongated dendrite-like processes with a bipolar arrangement; the nuclei were small and inconspicuous. No CD34 reactivity was observed in epithelial cells. SMA was detected in the wall of muscular vessels and in myoepithelia lining the ductal and acinar basement membranes, whereas SMA-reactive myofibroblasts were not detected in the stroma of normal breast tissue (Fig. 1).

### Ductal Carcinoma in situ

The most variable pattern of expression was seen in fibroblasts surrounding ducts with DCIS. A statistically significant difference in CD34 and SMA expression was found depending on the grade of DCIS. Indeed, loss of CD34 expression and acquisition of SMA expression were more frequent in high-grade lesions than in intermediate lesions or low-grade lesions (p<0.001). In high grade DCIS, fibroblast CD34 expression was reduced in 87% of cases compared with 20% of intermediate and 0% of low-grade cases of DCIS (Table 2; Fig. 1). In most cases, loss of CD34 expression was accompanied by the acquisition of SMA expression, and this was also significantly more frequent in high nuclear grade DCIS compared with intermediate and low grade DCIS (p<0.001). However, we did not observe any difference in the expression of CD34 ans SMA in cases of DCIS, located at the periphey of IDC compared to cases of pure DCIS without IDC.

**Table 2:** Summary of CD34 and SMA expression patterns in stroma according to histopathological features.

Histology	CD34 fibrocytes			SMA myofibroblasts		
	+	−	+/−	+	−	+/−
Normal	300	0	0	0	300	0
Low-grade DCIS	117	0	0	1	44	53
Intermediate-grade DCIS	206	12	40	8	33	159
High-grade DCIS	13	11	152	14	76	86
IDC	0	28	0	23	1	4

DCIS: ductal carcinoma in situ; IDC: invasive ductal carcinoma; SMA: alpha smooth muscle antigen.

In contrast with normal mammary tissue, the number of CD34 fibrocytes was reduced in the stroma surrounding ducts harboring carcinoma in situ, although capillaries with CD34 reactive endothelia were still detectable adjacent to these ducts. In contrast, ducts with DCIS were encircled by a rim of densely packed SMA myofibroblasts, which were not detected in normal breast tissue.

### Role of Necrosis

We assessed whether the presence of necrosis associated with grade 2 or 3 in situ lesions could influence the expression of CD34 and SMA. When necrosis was present, the decrease in CD34 expression was higher than in lesions without necrosis and that independently of the grade of DCIS (p<0.05). However, necrosis did not appear to play a significant role in SMA expression (p = 0.35).

### Invasive Ductal Carcinoma

In all cases investigated (100%), the stroma of invasive carcinomas showed a complete loss of CD34 fibrocytes, while the surrounding mammary tumor-free tissue disclosed a normal distribution of this cell population (Fig. 1). Twenty-seven of 28 invasive ductal carcinomas revealed SMA myofibroblasts forming focal accumulations of various extent. No difference was found between the different grades or regarding luminal classification. In addition, the SMA expression was present regardless of the type of the stroma observed within IDC (sparsely cellular hyalinised to more cellular desmoplastic stroma). When in situ lesions were located in the center of invasive areas, they tended to present less intense CD34 expression, while also showing higher expression of SMA, but to a lesser extent.

## Discussion

The DCIS to IDC transition is a clinically important, yet poorly understood, step in breast tumorigenesis. The importance of changes in the microenvironment during tumor progression has been increasingly recognized [2], [3], [11]. CD34 is a transmembrane glycoprotein expressed by hematopoietic stem cells, endothelial cells and mesenchymal cells in different tissues, including breast tissue, and is thought to be involved in the modulation of cell adhesion and signal transduction. They constitute a population of stromal cells that are capable of synthetizing connective tissue matrix. Moreover, CD34 fibrocytes are potent antigen-presenting cells and they may play a role in host response to tissue damage [4], [12]–[14]. However, their histogenesis and function have not yet been elucidated. Some authors have suggested that CD34 fibrocytes in breast lesions are recruited via the bloodstream and invade sites of tissue damage [8]. We believe that the CD34 fibrocytes altered (transformed into SMA myofibroblasts) by tissue damage are fibrocytes already present in stroma (in a constitutional manner) before the onset of lesions, and no recruitment of CD34 fibrocytes from the blood occurs, at least in breast tissue. This hypothesis relies on the facts that CD34 stromal cells are already present from the 10^th^ week of gestation and that there is strong expression of CD34 fibrocytes in the normal breast. This distribution of CD34 fibrocytes is similar to that of normal skin, and we know that the mammary gland develops from solid epithelial cords growing from the epidermal layer into the underlying mesenchyme [15], and therefore has the same embryological characteristics as normal skin.

Several studies have shown the loss of CD34 fibrocytes to be a feature of stromal alterations associated with invasive carcinomas of the breast [8]–[10] (Table 3). This is underlined by the findings of the present study. Invasive carcinomas revealed complete loss of stromal CD34 fibrocytes in 100% of cases. Loss of CD34 expression was accompanied by acquisition of SMA expression, with only a few exceptions. These data indicate a strong negative association between the presence of CD34 fibrocytes and the malignancy of ductal breast lesions. However, the stroma surrounding ductal carcinoma in situ was also characterized by loss of CD34 fibrocytes. Indeed, we found a decrease in CD34 expression in DCIS lesions compared with normal breast tissue. This loss of expression had a stronger effect when the grade of DCIS was higher. These results were in accordance with those of Chauhan et al. [10]. Furthermore, this decrease in CD34 expression was inversely correlated with a progressive increase in the expression of SMA around foci of DCIS. To our knowledge, this is the first time that a statistically significant difference in the expression of SMA in relation to DCIS grade has been demonstrated. Similarly, this is one of the first studies to demonstrate a statistically significant relationship between the necrosis associated with in situ lesions and immunohistochemical stromal expression of CD34. Indeed, several studies have shown that comedonecrosis on core biopsy is significantly associated with invasion [16]–[18]. The various factors secreted during necrosis could decrease the expression of CD34 stroma, facilitating tumor invasion. Therefore, modulation of the expression of CD34 could be one explanation of the role of necrosis in tumor invasion.

**Table 3:** Expression of CD34 fibrocytes and SMA myofibroblasts in DCIS and IDC: literature review.

	Barth, 2002		Cimpean, 2005		Chauhan, 2003		Catteau	
	CD34	SMA	CD34	SMA	CD34	SMA	CD34	SMA
Normal	100%	0%	100%	0%	100%	0%	100%	0%
DCIS	0%	71%	0%	100%	Low: 65%	Low: 73%	Low: 100%	Low: 55%
					Inter: 62%	Inter: 96%	Inter: 95%	Inter: 83.5%
					High: 22%	High: 100%	High: 94%	High: 57%
IDC	0%	83%	0%	100%	0%	100%	0%	82%

DCIS: ductal carcinoma in situ; IDC: invasive ductal carcinoma; SMA: alpha smooth muscle antigen; Inter: intermediate.

However, the mechanism leading to the loss of CD34 fibrocytes in the stroma of carcinomas are far from being understood. Breast cancer cells have been shown to be capable of factor secretion [19]. Therefore, we speculate that loss of CD34 fibrocytes and gain of SMA myofibroblasts might be initiated by a soluble factor secreted by DCIS cells. It has been shown that medium conditioned with breast cancer cell line MCF-7 induces SMA expression in stromal cells obtained from normal breast tissue [20]. Moreover, some research has found that CD34 fibrocytes acquire SMA expression when exposed to transforming growth factor-β [21], [22]. Considering the results of the present study, it appears to be more likely that CD34 fibrocytes acquire SMA expression and in turn down-regulate CD34 expression.

We believe that several mechanisms may explain the promotion of tumor invasion that induces the transformation of CD34 fibrocytes to SMA myofibroblasts. First, CD34 fibrocytes are potent antigen-presenting cells capable of priming naïve T-cells, and might be involved in specific immune surveillance [23], [24]. A subpopulation of CD34 fibrocytes expresses MHC II molecules and CD80 [25]. Second, CD34 fibrocytes are involved in the remodeling of stromal tissue damage not only via tissue contractility via TGF-beta, collagen I and III synthesis and SMA, but also in terms of migration factors within the injured tissue via CCR7, CXCR4, SLC, and CXCL12. Third, CD34 fibrocytes also play a role in angiogenesis via bFGF, VEGF, PDGF-a, IL-8, and MMP-9. The stromal reaction induced by carcinomatous lesions leads to acquisition of SMA expression and in turn to stabilization of the lesion (wound contraction) that helps prevent the spread of tissue damage [26]. This may reflect a defense mechanism against “stromal invasion” that induces a phenomenon of stromal healing and stabilization. However, the phenotypic transformation of CD34 fibrocytes into SMA myofibroblasts could also cause the loss of most essential functions (including immunity, cell adhesion, motility, stromal remodeling, and angiogenesis inhibition), and in a paradoxical manner promote tumorigenesis, thus facilitating invasion and metastatic dissemination of tumor cells. Our study has shown that fibroblast CD34 expression is consistently lost in invasive breast carcinomas, and in a high proportion of cases of DCIS, particularly necrotic and/or high-grade lesions, which are thought to be more likely to progress to invasion [16], [27]. Moreover, the change in CD34 expression is very localized, with loss around ducts containing DCIS, but retained expression around adjacent normal breast glands [12]. This raises the possibility that loss of CD34 may be related to invasive potential at least in ductal lesions. Indeed, in invasive lobular carcinoma that tend not to be associated with an altered stroma, our preliminary observations do not confirm so obviously the stromal loss of CD34 and acquisition of SMA (unpublished data).

Therefore, future research on both CD34 fibrocytes and mechanisms stromal changes are essential in the future and may potentially lead to new treatment approaches. Such studies are now in progress.
